# BG-YOLO11s: Boundary-Guided YOLO11 with Bézier Contour Augmentation for Brain Tumour Segmentation in T1-CE MRI

**DOI:** 10.3390/diagnostics16152407

**Published:** 2026-07-30

**Authors:** Mustafa Yurdakul, Javanshir Zeynalov, Merve Ersoy, Faruk Özger, Ishak Pacal

**Affiliations:** 1 Department of Computer Engineering, Kırıkkale University, Kırıkkale 71450, Türkiye; 2 Department of Electronics and Information Technologies, Faculty of Architecture and Engineering, Nakhchivan State University, AZ 7012 Nakhchivan, Azerbaijan; cavansirzeynalov@ndu.edu.az; 3 Department of Computer Engineering, İstanbul Topkapı University, İstanbul 34394, Türkiye; merveersoy@topkapi.edu.tr; 4 Department of Computer Engineering, Iğdır University, Iğdır 76000, Türkiye; faruk.ozger@igdir.edu.tr (F.Ö.); ishak.pacal@igdir.edu.tr (I.P.); 5 Department of Computer Engineering, Faculty of Engineering and Natural Sciences, Fenerbahce University, Istanbul 34758, Türkiye

**Keywords:** brain tumour, MRI, instance segmentation, YOLO11, Bézier curve, data augmentation, deep learning, medical image analysis

## Abstract

**Background/Objectives:** Accurate delineation of brain tumours on contrast-enhanced MRI remains difficult because lesions can be small, irregular, and weakly separated from adjacent tissue. This study developed BG-YOLO11s, a boundary-guided single-stage instance-segmentation model for T1 contrast-enhanced MRI. **Methods:** The public Figshare/Cheng dataset, comprising 3064 slices from 233 patients, was converted to YOLO polygon annotations and evaluated using a fixed 70/15/15 image-level split (2144/459/461 slices). Because patient identifiers were not retained in the exported image-and-polygon data, the split was not guaranteed to be patient-disjoint. Bézier Contour Augmentation generated two contour-perturbed training samples per original slice while leaving validation and test data unchanged. BG-YOLO11s extended YOLO11s-seg with dilated context aggregation in the backbone, boundary-enhanced feature fusion in the neck, and a prototype refinement module with differentiable boundary-aware supervision in the segmentation head. **Results:** In a single run on the held-out image-level test split, BG-YOLO11s achieved 92.4% precision, 88.7% recall, 94.6% mask mAP@50, 68.9% mAP@50–95, and 86.5% IoU. Relative to YOLO11s-seg, the corresponding gains were 3.8 points in mAP@50, 5.8 points in mAP@50–95, and 4.1 points in IoU. A progressive ablation produced incremental gains along the fixed module-addition sequence, but it did not isolate all component interactions or quantify run-to-run uncertainty. **Conclusions:** BG-YOLO11s improved single-run mask-overlap estimates under the present image-level benchmark. Patient-disjoint retraining, repeated-seed statistics, boundary-specific metrics, complete failure pattern auditing, and external multi-sequence validation are required before broader clinical or deployment claims can be made.

## 1. Introduction

Brain tumours represent a heterogeneous group of intracranial lesions with different biological behaviour, anatomical distribution, and clinical consequences [1]. The 2021 World Health Organization classification further emphasises their molecular and histopathological diversity [2]. Their clinical impact depends not only on histological type and malignancy, but also on lesion location, growth pattern, mass effect, and the extent of surrounding tissue involvement. Magnetic resonance imaging is the main modality for brain tumour assessment because it provides high soft-tissue contrast and allows detailed evaluation of tumour extent without ionising radiation. Among routine MRI sequences, contrast-enhanced T1-weighted imaging is particularly useful for identifying enhancing tumour tissue, especially when disruption of the blood–brain barrier produces visible but irregular contrast uptake.

Although brain and central nervous system tumours are less frequent than many other cancers, their diagnostic and prognostic burden remains substantial. Recent CBTRUS surveillance documents important differences in incidence and survival between malignant and non-malignant primary central nervous system tumours [3]. Earlier CBTRUS reporting similarly characterised the epidemiological burden of these tumours [4]. Global cancer estimates also indicate a considerable disease burden worldwide [5]. Reliable tumour delineation on MRI is, therefore, important for lesion quantification, treatment planning, follow-up assessment, and computational analysis. In routine practice, however, manual or semi-manual segmentation is still widely used and remains time-consuming, expertise-dependent, and sensitive to intra-observer and inter-observer variability.

The public Figshare/Cheng brain tumour dataset has become a widely used benchmark for T1 contrast-enhanced MRI analysis. It contains 3064 slices from 233 patients and includes meningioma, glioma, and pituitary tumour cases acquired in axial, coronal, and sagittal planes. The dataset is useful for segmentation research because it provides tumour masks together with clinically relevant variation in tumour type, imaging plane, lesion size, and boundary appearance. At the same time, the task is difficult because many tumours occupy a small image area, while their enhancing margins may be weak, irregular, or partially blended with adjacent tissue.

Classical segmentation methods, such as thresholding, region growing, clustering, and active contours, are computationally simple but fragile under intensity inhomogeneity, partial-volume effects, weak boundaries, and variable tumour morphology. U-Net established a strong skip-connected encoder–decoder framework for dense biomedical image segmentation [6]. An improved U-Net with hybrid dilated convolutions and a Dice-based objective further demonstrated the value of multi-scale context for brain tumour segmentation [7]. More recent approaches have introduced attention mechanisms, transformer-based modelling, and hybrid CNN-Transformer designs to improve contextual representation and boundary localisation. Despite these advances, many high-capacity segmentation networks remain relatively complex and are not always designed for compact single-stage inference.

YOLO-based models offer a different formulation by combining localisation and mask prediction within a single forward pass. The original YOLO framework was developed for real-time object detection [8]. Prototype-based real-time instance segmentation was subsequently demonstrated by YOLACT [9]. Recent descriptions of YOLO11 report updated feature extraction and multi-scale representation [10]. The experiments in this study use the Ultralytics YOLO11 implementation [11]. This model family is, therefore, a promising candidate for compact medical image segmentation. However, direct use of a generic YOLO segmentation model may be insufficient for brain tumour masks, where small lesion size and weak boundary contrast can limit both localisation and contour precision.

Recent brain tumour segmentation research includes several architectures directly relevant to this work. YOLO-BT targeted irregular tumour shape and size variation in MRI segmentation [12]. A separate benchmark compared U-Net, FCN, and multiple YOLO variants on the BTS-DS 2024 dataset [13]. A two-headed U-Net-EfficientNet model combined segmentation and classification [14]. QuickTumorNet explored fast multi-class tumour segmentation [15]. A two-stage U-Net and DenseNet pipeline combined lesion localisation with tumour-type prediction [16]. Volumetric 3D convolutional neural networks have also been used to exploit inter-slice MRI context [17]. The BraTS 2024 glioma challenge emphasised patient-disjoint, multi-institutional, multi-sequence evaluation with lesion-wise boundary measures [18]. The BraTS-METS benchmark applied a similarly rigorous framework to pre-treatment brain metastasis segmentation [19]. Together, these studies show that multi-scale context, inter-slice information, multi-task learning, and single-stage prediction are useful for handling tumour variability, but explicit boundary modelling and contour-level augmentation are less frequently examined together. Standard geometric and photometric augmentation can increase image diversity, yet it does not directly enrich the shape variability of tumour contours.

To address these issues, this study proposes BG-YOLO11s, a boundary-guided instance-segmentation model built on YOLO11s-seg for brain tumour delineation in contrast-enhanced T1-weighted MRI. The model introduces DCA to improve contextual coverage for lesions with variable spatial extent, BEFF to strengthen edge-sensitive features, and a PRM with boundary-aware supervision to improve mask quality. In addition, Bézier Contour Augmentation perturbs tumour boundaries during training, increasing contour-level variability while preserving the surrounding anatomical context. The model is evaluated on the Figshare/Cheng dataset using YOLO11 segmentation baselines and a progressive module-addition analysis. The main contributions of this study are as follows:

- A boundary-guided YOLO11-based instance-segmentation framework is proposed for brain tumour mask delineation in contrast-enhanced T1-weighted MRI.
- DCA is integrated into the backbone to improve contextual representation for small and size-variable tumour regions.
- BEFF and a PRM with boundary-aware supervision are introduced to improve mask precision around weak and irregular tumour margins.
- Bézier Contour Augmentation is developed as a contour-level training strategy that increases tumour shape variability without changing distant anatomical structures.
- The proposed model is evaluated on the public Figshare/Cheng dataset against YOLO11 segmentation baselines, and a progressive ablation quantifies incremental gains under a fixed module-addition order. The design does not constitute a full factorial analysis of every module combination.

## 2. Related Works

Brain tumour segmentation in contrast-enhanced MRI has been studied mainly through encoder–decoder networks, attention-based architectures, multi-task pipelines, and more recently single-stage segmentation models. The Figshare/Cheng dataset has played an important role in this literature because it provides tumour masks for T1 contrast-enhanced MRI slices and has been used repeatedly for both segmentation and classification studies. Earlier segmentation work on this dataset has shown that tumour delineation is affected by lesion size, enhancement heterogeneity, and boundary ambiguity, which remain relevant even when modern deep learning models are used.

U-Net-based models remain a common starting point for brain tumour segmentation because their skip-connected encoder–decoder structure preserves spatial detail while learning semantic representation [6]. Zheng et al. improved U-Net with hybrid dilated convolutions and a Dice-based objective to capture tumour context at multiple scales [7]. These works establish the importance of spatial reconstruction and multi-scale context, but they do not directly examine contour-level augmentation or boundary-aware prototype refinement.

The YOLO family introduced single-stage object detection within one forward pass [8]. YOLACT later demonstrated efficient instance segmentation through shared prototype masks and instance-specific coefficients [9]. YOLO11 incorporates updated feature extraction and multi-scale design choices [10]. The Ultralytics implementation provides the segmentation framework used as the baseline in this study [11].

Recent YOLO-based work has begun to adapt this family to brain tumour segmentation. Xiong et al. proposed YOLO-BT to address irregular tumour shape and size variation in MRI [12]. A separate study compared U-Net, FCN, and several YOLO variants on the BTS-DS 2024 dataset [13]. These findings support the potential of compact single-stage models, although generic segmentation heads may still be limited when lesions are small or have diffuse margins.

Multi-task and multi-class architectures provide complementary approaches. A two-headed U-Net-EfficientNet model performed segmentation and classification in parallel with connected-component post-processing [14]. QuickTumorNet focused on fast multi-class tumour segmentation across different imaging planes [15]. Another pipeline used a U-Net with a ResNet-18 encoder for lesion localisation followed by a DenseNet classifier for tumour-type prediction [16]. Such designs improve clinical task coverage, but they often rely on encoder–decoder, multi-task, or multi-stage processing rather than a compact boundary-guided instance-segmentation pipeline.

Volumetric and multi-sequence methods provide complementary evidence regarding generalisation and boundary evaluation. Ramzan et al. used 3D convolutional neural networks for volumetric segmentation of brain regions from MRI [17]. The BraTS 2024 glioma challenge uses patient-disjoint, multi-institutional, multi-sequence cohorts and lesion-wise boundary measures [18]. The BraTS-METS benchmark applies related evaluation principles to pre-treatment brain metastasis segmentation [19]. These benchmarks are not directly comparable with the present 2D T1-CE task, but they establish a stronger standard for patient-level, external, multi-sequence, and boundary-specific validation.

Weakly supervised and explainability-oriented segmentation has also been explored for brain tumour analysis. Inherently explainable classification models have been used to obtain segmentation evidence without dense pixel-level supervision [20]. Although such approaches reduce annotation dependence, they usually provide less direct control over boundary precision than fully supervised mask-based segmentation.

In a complementary direction, Yurdakul et al. proposed region-wise Bézier intensity augmentation within a Mamba U-Net for domain-generalised brain tumour segmentation [21]. That strategy enriches intensity variability across imaging domains, whereas the present Bézier Contour Augmentation directly perturbs tumour contour geometry while preserving distant anatomical structures. Across the reviewed studies, multi-scale representation, attention, volumetric context, and single-stage prediction are consistently useful, but explicit contour-level augmentation and boundary-refined prototype masks are rarely examined together. These observations motivate BG-YOLO11s while also defining the limits of the current single-dataset evaluation.

The present study addresses this gap by developing BG-YOLO11s for tumour mask segmentation in contrast-enhanced T1-weighted MRI. Unlike standard YOLO11s-seg, the proposed model adds dilated context aggregation, boundary-enhanced feature fusion, and a prototype refinement module with boundary-aware supervision. In addition, Bézier Contour Augmentation is used to increase tumour shape variability during training. This combination is intended to improve both lesion localisation and contour precision while retaining the compact structure of a single-stage segmentation framework. Table 1 summarises representative brain tumour segmentation studies conducted on the Figshare/Cheng dataset or on closely related T1-CE MRI data.

**Table 1:** Representative brain tumour segmentation studies using the Figshare/Cheng dataset or closely related T1-CE MRI data.

Study	Dataset	Task	Model Category	Main Methodological Focus	Reported Performance	Limitation Relevant to This Study
Zheng et al. [7]	Figshare/Cheng	Tumour segmentation	Improved U-Net	Hybrid dilated convolutions and Dice-based optimisation for multi-scale tumour context	Improved Dice over vanilla U-Net	Boundary modelling and contour-level augmentation are not explicitly evaluated
Xiong et al. [12]	T1-CE brain MRI	Tumour segmentation	YOLO-based segmentation	YOLOv11-based model designed for irregular tumour shape and size variation	mIoU +6.1%, Dice +3.6% over baseline	Does not jointly examine YOLO11 segmentation with explicit contour-level augmentation and boundary-refined prototype masks
U-Net/FCN/YOLO hybrid study [13]	BTS-DS 2024	Tumour segmentation	CNN and YOLO benchmarking	Comparative evaluation of U-Net, ResU-Net, FCN, YOLOv8, YOLOv9, and YOLO11 variants	YOLOv9e: F1 92.25%, IoU 85.62%	Evaluated on a different dataset, so results are not directly comparable with Figshare/Cheng
Two-headed U-Net-EfficientNet [14]	Figshare/Cheng	Segmentation and classification	Multi-task encoder–decoder	Parallel tumour segmentation and classification with connected-component post-processing	NR	Uses a multi-stage or multi-task design rather than a single-stage segmentation pipeline
QuickTumorNet [15]	Cheng 2D MRI slices	Multi-class tumour segmentation	Fast CNN-based segmentation	Fast segmentation across axial, coronal, and sagittal slices	NR	Emphasises speed and multi-class segmentation but does not explicitly model tumour boundary refinement
U-Net with ResNet-18 and DenseNet [16]	Figshare/Cheng	Segmentation followed by classification	Two-stage CNN pipeline	U-Net localisation with ResNet-18 encoder followed by DenseNet classification	Classification accuracy approximately 91–97% per tumour class after segmentation	Segmentation is used as a localisation stage; boundary-specific mask optimisation is not the main focus
Ramzan et al. [17]	Volumetric brain MRI	Brain region segmentation	3D CNN	Volumetric segmentation using inter-slice context	Reported in the original study; metrics are not directly comparable with the present tumour task	Not specific to single-stage T1-CE tumour instance segmentation and requires complete 3D volumes
BraTS glioma challenge [18]	Multi-institutional, multi-sequence MRI	Glioma segmentation	Challenge benchmark	Patient-disjoint evaluation using lesion-wise Dice and Hausdorff distance measures	Challenge-specific rankings and metrics	Different cohorts and targets, but a stronger standard for external and boundary-sensitive validation
Weakly supervised XAI study [20]	Cheng/BraTS	Weakly supervised tumour localisation and segmentation	Explainability-based segmentation	Inherently explainable classifier used to derive segmentation evidence without dense mask supervision	NR	Reduces annotation dependence but provides less direct control over boundary precision than fully supervised mask training
BG-YOLO11s, this study	Figshare/Cheng	Single-class tumour instance segmentation	Boundary-guided YOLO11 segmentation	DCA, BEFF, PRM with boundary-aware supervision, and Bézier Contour Augmentation	mAP@50 94.6%, mAP@50–95 68.9%, IoU 86.5%	Current evaluation uses an image-level split; patient-disjoint and external validation are still required

## 3. Materials and Methods

All experiments were conducted on the public Figshare/Cheng brain tumour dataset using a single-class instance-segmentation formulation. The workflow consisted of four main stages: conversion of binary tumour masks into YOLO-compatible polygon annotations, contour-level augmentation of training masks, training of YOLO11-based segmentation models, and evaluation on the held-out test set. The proposed model, BG-YOLO11s, was developed by modifying YOLO11s-seg with context, boundary, and prototype refinement components.

### 3.1. Dataset

**Figure 1:**
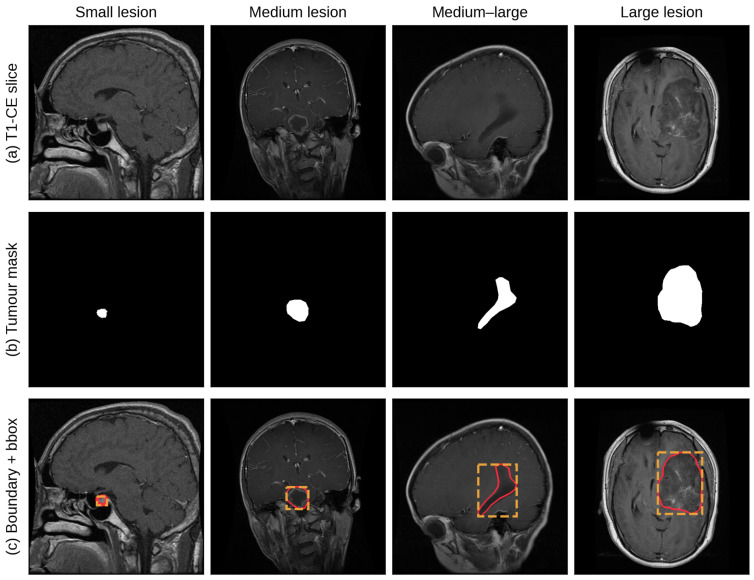
Representative contrast-enhanced T1-weighted MRI slices from the Figshare/Cheng dataset. The examples cover small, medium, and large tumour regions across axial, coronal, and sagittal planes. (**a**) Input T1-CE slice; (**b**) binary tumour mask; (**c**) tumour boundary and bounding box overlaid on the input slice, where the **red solid line** denotes the delineated tumour contour and the **orange dashed rectangle** denotes the corresponding bounding box.

All experiments were conducted on the public Figshare brain tumour dataset originally described by Cheng et al. [22]. The corresponding dataset release is available through Figshare [23]. The dataset contains 3064 contrast-enhanced T1-weighted MRI slices from 233 patients and includes 3 tumour types: meningioma, glioma, and pituitary tumour. The images were acquired at Nanfang Hospital, Guangzhou, and the General Hospital of Tianjin Medical University, China. Each slice is provided with a manually delineated tumour mask, which allows supervised evaluation of tumour segmentation models [22]. Representative examples from different lesion sizes and imaging planes are shown in Figure 1.

The dataset contains 708 meningioma, 1426 glioma, and 930 pituitary tumour slices. The images cover 3 anatomical planes, with 994 axial, 1045 coronal, and 1025 sagittal slices. In the present study, the task was formulated as single-class tumour delineation rather than histological classification. Therefore, all tumour masks were treated as one foreground class, denoted as tumour. The composition and acquisition characteristics of the dataset are summarised in Table 2.

**Table 2:** Composition and acquisition characteristics of the Figshare/Cheng brain tumour dataset.

Attribute	Value
Modality	Contrast-enhanced T1-weighted MRI
Contrast agent	Gd-DTPA
Total slices/patients	3064/233
Image resolution	512 × 512 pixels
Pixel spacing	0.49 × 0.49 mm^2^
Slice thickness/gap	6 mm/1 mm
Tumour types	Meningioma, glioma, pituitary tumour
Meningioma/glioma/pituitary	708/1426/930 slices
Axial/coronal/sagittal	994/1045/1025 slices
Mean tumour area	1.69% of image area
Median tumour area	1.29% of image area
Small lesions	1243 slices, <1% of image area
Medium lesions	1718 slices, 1–5% of image area
Large lesions	103 slices, >5% of image area
Single-component masks	3029 slices
Multi-component masks	35 slices
Train/validation/test split	2144/459/461 slices
Split ratio	70/15/15
Segmentation class	Single foreground class, tumour

**Figure 2:**
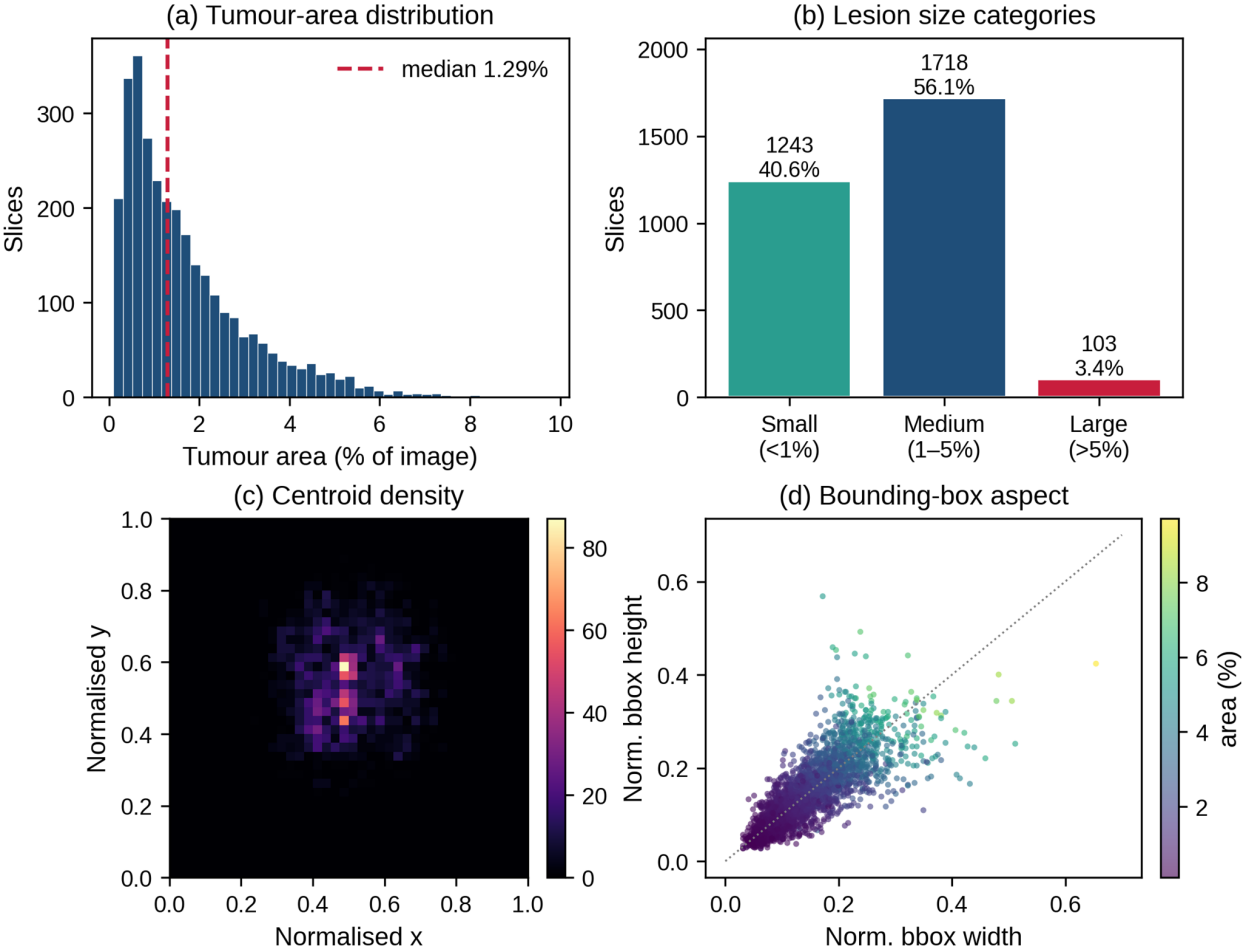
Dataset statistics computed from the tumour masks: (**a**) relative tumour area distribution, (**b**) lesion size categories, (**c**) tumour-centroid spatial density, and (**d**) bounding box aspect ratio distribution.

Mask-level analysis showed that most tumours occupied only a small fraction of the image. The mean tumour area was 1.69% of the image area, and the median was 1.29%. Based on relative tumour area, 1243 lesions were smaller than 1%, 1718 were between 1% and 5%, and only 103 exceeded 5% of the full image. Most masks consisted of a single connected component, while 35 slices contained more than 1 component. These statistics indicate that the benchmark is dominated by small and medium-sized lesions with variable morphology, which motivated the context-aware and boundary-sensitive design of the proposed model. The lesion size distribution, centroid density, and bounding box characteristics are summarised in Figure 2.

The dataset was divided into training, validation, and test subsets using a fixed 70/15/15 image-level split, corresponding to 2144, 459, and 461 slices, respectively. The same split was used for all baseline and proposed models to ensure protocol consistency. The exported image-and-polygon dataset used for the experiments did not preserve patient identifiers; therefore, the split could not be guaranteed to be patient-disjoint. Reconstructing patient groups from the original MAT-file release and retraining every baseline and ablation configuration would be required for a patient-level analysis. Accordingly, the reported values are image-level benchmark results and should not be interpreted as evidence of generalisation to unseen patients.

**Figure 3:**
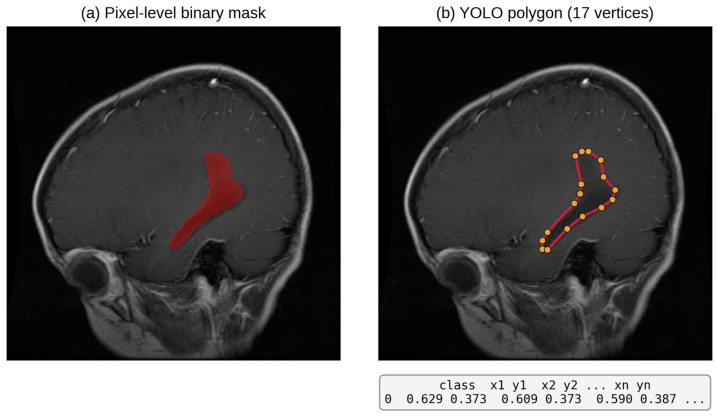
Conversion of binary tumour masks into YOLO instance-segmentation format. (**a**) Pixel-level binary tumour mask, shown in **red** overlaid on the T1-CE slice; (**b**) resulting YOLO polygon, in which the **red solid line** is the simplified tumour contour and the **orange markers** are the polygon vertices written to the annotation file. External tumour contours are extracted from the binary mask, simplified into polygon vertices, normalised to image coordinates, and written as class-labelled segmentation annotations.

For YOLO-based instance segmentation, each binary tumour mask was converted into polygon annotations. External contours were extracted from the binary masks and simplified using polygonal approximation. Each polygon was then written in normalised YOLO segmentation format, where the first value denotes the class index and the remaining values denote x and y vertex coordinates normalised by image width and height. For multi-component masks, each valid connected component was converted into a separate polygon under the same tumour class. This avoided merging disconnected tumour regions into a single artificial contour. The conversion procedure is illustrated in Figure 3.

### 3.2. Bézier Contour Augmentation

Conventional augmentation strategies, such as flipping, rotation, scaling, cropping, and intensity perturbation, increase image diversity but leave the intrinsic morphology of the tumour boundary largely unchanged. This is a limitation for brain tumour segmentation because mask quality is often determined by small boundary deviations, especially when the enhancing margin is weak or irregular. To increase contour-level variability during training, this study introduces Bézier Contour Augmentation, a shape-aware augmentation strategy that locally perturbs tumour boundaries while preserving the surrounding anatomical context.

Smooth nonlinear intensity transformations have been used to support representation learning in medical imaging [24]. Bézier curves have also been used for compact parametric shape representation in medical segmentation [25]. Augmentation design has further been shown to influence single-source domain generalisation in medical image segmentation [26]. In the proposed augmentation, each tumour component is represented as a closed contour and deformed through controlled low-frequency radial perturbations. Given a binary mask, the external contour of each valid tumour component is extracted. The centroid of the component is computed, and the contour points are expressed in polar coordinates around this centroid. The boundary is then resampled into K angular anchors to obtain a radial profile r(θ).

The radial perturbation is defined as:

(1) $$\delta \left(\theta \right)=\alpha \;\frac{\sum_{k=1}^{m}a_{k}sin\left(k\theta +\phi_{k}\right)}{\underset{\vartheta }{max}\left|\sum_{k=1}^{m}a_{k}sin\left(k\vartheta +\phi_{k}\right)\right|+\varepsilon },$$

where α controls the maximum relative displacement of the contour, m denotes the number of low-frequency modes, and a_k_ and φ_k_ are randomly sampled amplitude and phase parameters. In the denominator, ϑ is a dummy angular variable and the maximisation is taken over ϑ ∈ [0, 2π). A numerical stability constant ε = 10^−6^ is added to the denominator. If the unnormalised sinusoidal perturbation is identically zero, the numerator is also zero and δ(θ) remains zero, so the original contour is retained. The perturbed radial profile is then obtained as:

(2) $$r\prime \left(\theta \right)=r\left(\theta \right)\left[1+\delta \left(\theta \right)\right].$$

This formulation restricts the deformation to smooth, low-frequency shape changes and avoids unrealistic high-frequency distortions along the tumour boundary. The perturbed radial profile is converted back to Cartesian coordinates and interpolated as a smooth closed curve. For a cubic Bézier segment with control points $P_{0},P_{1},P_{2},$ and $P_{3}$, the curve is written as:

(3) $$B\left(t\right)={\left(1-t\right)}^{3}P_{0}+3{\left(1-t\right)}^{2}tP_{1}+3\left(1-t\right)t^{2}P_{2}+t^{3}P_{3},\;0\leq t\leq 1.$$

The augmented mask is generated by filling the perturbed closed contour. To avoid unrealistic global deformation of the MRI slice, the image transformation is constrained to the lesion neighbourhood. Corresponding points are sampled from the original and perturbed contours, and additional anchors are placed around the lesion and along the image frame. These anchors stabilise the transformation outside the tumour region and preserve distant anatomical structures. The local image deformation and regenerated mask can be expressed as:

(4) $$I\prime \left(p\right)=I\;\left(T^{-1}\left(p\right)\right),\;\;M\prime =fill\left(C\prime \right),$$

where I and $I^{\prime }$ are the original and augmented images, T is the local piecewise-affine transformation, $C^{\prime }$ is the perturbed tumour contour, and $M^{\prime }$ is the regenerated binary mask.

**Figure 4:**
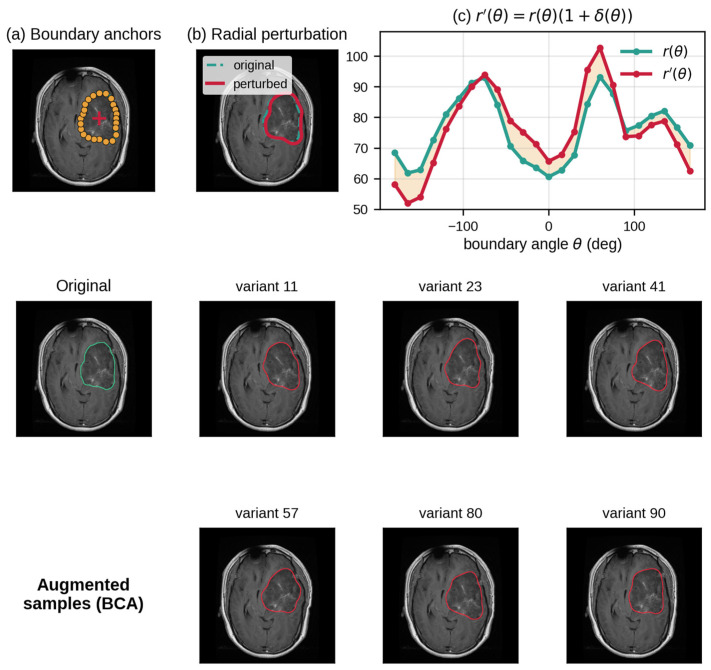
Bézier Contour Augmentation. (**a**) Boundary anchors: the **orange markers** are the K angular anchors sampled around the lesion and the **red marker** is the component centroid; (**b**) radial perturbation: the **green dashed line** is the original contour r(θ) and the **red solid line** is the perturbed contour r′(θ); (**c**) corresponding radial profiles, where the **green curve** is r(θ), the **red curve** is r′(θ), and the shaded area indicates the applied radial displacement. The lower rows show the original slice (**green contour**) and representative augmented variants (**red contours**). The deformation is localised around the tumour region to increase contour variability while preserving surrounding anatomical structures.Augmentation. The tumour boundary is represented by angular anchors around the lesion centroid, perturbed with smooth low-frequency radial offsets, and reconstructed as a closed contour. The deformation is localised around the tumour region to increase contour variability while preserving surrounding anatomical structures.

Bézier Contour Augmentation was applied only to the training split. The validation and test sets were kept unchanged. For each original training slice, two augmented samples were generated, increasing the number of training images from 2144 to 6432. The augmentation parameters were fixed before training and used consistently in all experiments involving BCA. This design allowed the ablation study to evaluate the contribution of contour-level augmentation separately from the architectural components of BG-YOLO11s. The augmentation workflow and representative deformed contours are shown in Figure 4.

### 3.3. YOLO Segmentation Models

The YOLO family formulates object detection as a single-stage prediction problem and combines localisation and classification within one forward pass [8]. YOLACT demonstrated efficient instance segmentation through prototype masks and instance-specific mask coefficients [9]. More recent YOLO implementations extend the same general principle by adding a mask branch to the detection architecture. The final mask for each detected object is obtained by combining shared prototype maps with the corresponding mask coefficients, allowing localisation and segmentation to be performed within the same architecture.

YOLO11 architectural refinements are described in recent overview work [10]. This study uses the Ultralytics YOLO11 implementation as the baseline segmentation framework [11]. YOLO11 follows the standard backbone, neck, and head design. The backbone extracts hierarchical visual features through convolutional blocks and spatial-pyramid aggregation. The neck fuses multi-scale features through top-down and bottom-up pathways, while the decoupled head predicts bounding boxes, class scores, and instance masks. This structure is suitable for brain tumour segmentation because it provides a compact prediction pipeline and allows the model to learn both lesion localisation and mask-level representation.

Three YOLO11 segmentation scales were evaluated as baselines: YOLO11n-seg, YOLO11s-seg, and YOLO11m-seg. These variants provide different capacity levels and allow the accuracy-parameter trade-off to be examined under the same dataset split and training protocol. The proposed model was built on YOLO11s-seg because the small variant provides a practical balance between model size and segmentation capacity. This choice also makes it possible to assess whether boundary-oriented architectural changes can improve segmentation quality without moving to a substantially larger backbone.

### 3.4. Proposed Model: BG-YOLO11s

**Figure 5:**
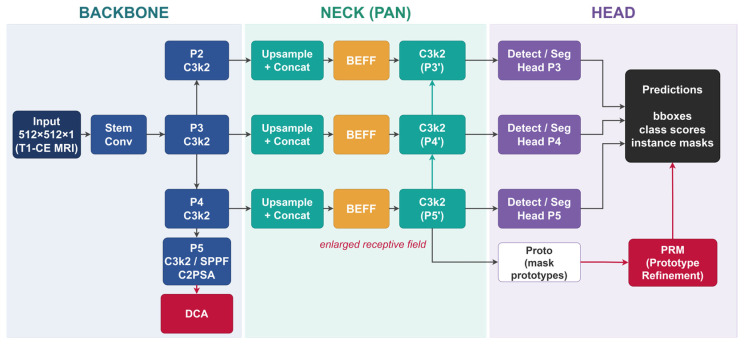
Architecture of the proposed BG-YOLO11s. The model extends YOLO11s-seg with dilated context aggregation in the backbone, boundary-enhanced feature fusion on the multi-scale neck outputs, and a prototype refinement module in the segmentation head. The model is trained with standard YOLO segmentation losses and an additional boundary-aware term.

BG-YOLO11s extends YOLO11s-seg with three task-specific components designed for small and irregular brain tumour masks. The first component, dilated context aggregation, is inserted into the backbone to enlarge contextual coverage. The second component, boundary-enhanced feature fusion, is applied in the neck to strengthen edge-sensitive features before prediction. The third component, the prototype refinement module, operates in the segmentation head and refines mask prototypes using boundary-aware supervision. The complete architecture is shown in Figure 5.

#### 3.4.1. Dilated Context Aggregation (DCA)

Brain tumour size varies considerably across the Figshare/Cheng dataset, with many lesions occupying only a small fraction of the image. A limited receptive field may miss broader anatomical context, while excessive downsampling can weaken small-lesion representation. To address this, dilated context aggregation was added after the high-level backbone feature extraction stage. The module aggregates information from multiple dilation rates while preserving the input feature resolution.

**Figure 6:**
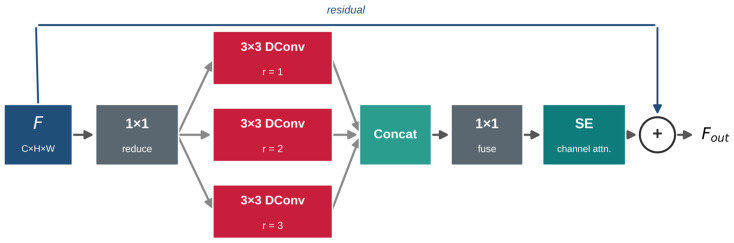
Dilated context aggregation module. The input feature is reduced by a 1×1 convolution, processed through parallel 3×3 dilated convolutions with dilation rates of 1, 2, and 3, fused by a 1×1 convolution, recalibrated with squeeze-and-excitation attention, and added back through a residual connection.

Given an input feature map F, the module first computes the reduced feature R = Conv_1_ × _1_red(F). Three parallel branches then produce D_r_ = DConv_3_ × _3_(r)(R) for r ∈ {1, 2, 3}. Their outputs are concatenated, fused by a 1 × 1 convolution, recalibrated by squeeze-and-excitation channel attention, and added to F through a residual connection [27]. The operation is defined as:

(5) $$F_{DCA}=F+SE\;\left({Conv}_{1\times 1}^{fuse}\;\left(Concat\left(D_{1},D_{2},D_{3}\right)\right)\right),$$

where Concat(·) denotes channel-wise concatenation, Conv_1_ × _1_fuse is the fusion convolution, and SE(·) denotes squeeze-and-excitation recalibration. The residual formulation preserves the input shape and reduces the risk of disrupting pretrained feature representations. The structure of the module is shown in Figure 6.

#### 3.4.2. Boundary-Enhanced Feature Fusion (BEFF)

Weak and irregular tumour margins are a common source of segmentation error in contrast-enhanced T1-weighted MRI. Standard multi-scale feature fusion improves semantic representation, but it does not explicitly emphasise boundary-aligned responses. Boundary-enhanced feature fusion was, therefore, applied to the neck outputs before the segmentation head. The module uses a learnable high-pass response to highlight local edge-sensitive activations and reweights the original feature accordingly.

For an input neck feature F, a high-pass response is computed as the difference between a depth-wise convolution and local average pooling:

(6) $$H\left(F\right)={DWConv}_{3\times 3}\left(F\right)-{AvgPool}_{3\times 3}\left(F\right).$$

The boundary attention map is then obtained using a 1×1 convolution followed by a sigmoid activation:

(7) $$A=\sigma \;\left({Conv}_{1\times 1}\left(H\left(F\right)\right)\right).$$

**Figure 7:**
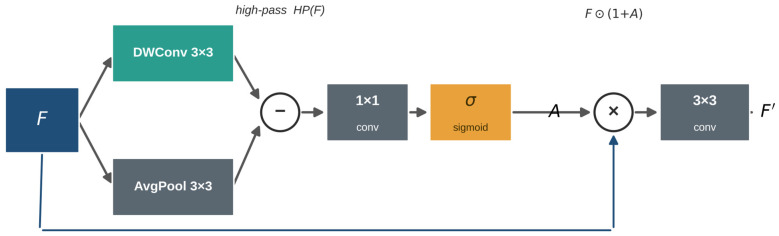
Boundary-enhanced feature fusion module. A learnable high-pass response is computed from the neck feature, converted into a boundary attention map, and used to reweight the original feature before the final convolution. The module is applied to the multi-scale neck outputs before mask prediction.

The input feature is reweighted using this attention map and passed through a 3×3 convolution:

(8) $$F_{BEFF}={Conv}_{3\times 3}\;\left(F⊙\left(1+A\right)\right),$$

where ⊙ denotes element-wise multiplication. This operation increases the contribution of boundary-sensitive activations while retaining the original semantic feature content. BEFF was applied to the three neck outputs used by the segmentation head. The module is illustrated in Figure 7.

#### 3.4.3. Prototype Refinement Module (PRM) and Boundary Loss

YOLO-style instance segmentation predicts a set of shared prototype maps and combines them with instance-specific coefficients to generate object masks [9]. This design is efficient, but fine boundary details may be weakened when small or irregular tumours are represented by a limited set of prototypes. To improve mask quality, a prototype refinement module was added to the segmentation head. The module applies a lightweight residual correction branch to the prototype maps before instance-mask reconstruction.

Let P denote the prototype maps produced by the segmentation head. The refined prototypes are computed as:

(9) $$\hat{P}=P+g\left(P\right),$$

where g(⋅) is a lightweight convolutional refinement branch. The refined prototypes $\hat{P}$ are then combined with the predicted mask coefficients to obtain the final instance mask $\hat{M}$. Boundary-aware supervision is applied to $\hat{M}$, not directly to the prototype maps, because the final instance mask is the object-specific prediction compared with the ground-truth mask.

The boundary term compares predicted and ground-truth mask boundaries using a differentiable morphological gradient operator. For a binary or probabilistic mask M ∈ [0,1], the operator is implemented as ∂(M) = MaxPool_3_ × _3_(M) − MinPool_3_ × _3_(M), where MinPool(M) = −MaxPool(−M). Both pooling operations use stride 1 and padding 1. No hard threshold is applied to the probabilistic prediction; therefore, the loss is evaluated on soft boundary maps. The same operator is applied to the binary ground-truth mask Y. The boundary-aware loss is defined as:

(10) $$L_{bnd}=BCE\;\left(\partial \left(\hat{M}\right),\partial \left(Y\right)\right),$$

where $\hat{M}$ is the post-sigmoid final predicted instance mask, Y is the corresponding ground-truth tumour mask, and BCE denotes binary cross-entropy. The loss is averaged over all pixels and all matched tumour instances in the batch. The final training objective combines the native YOLO segmentation losses with the boundary-aware term:

(11) $$L=L_{box}+L_{cls}+L_{dfl}+L_{mask}+\lambda_{b}L_{bnd},$$

where Lbox, Lcls, Ldfl, and Lmask are the standard YOLO bounding box, classification, distribution focal, and mask losses, respectively, and λb controls the contribution of the boundary term. This formulation encourages the final predicted mask to align with the ground-truth boundary while preserving the original YOLO segmentation objective.

**Figure 8:**
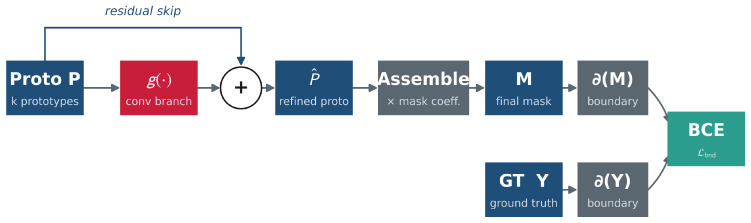
Prototype refinement module and boundary-aware supervision. Prototype maps produced by the segmentation head are refined through a residual convolutional branch and then combined with instance-specific coefficients to obtain the final mask. Boundary-aware loss is applied to the final predicted instance mask and the corresponding ground-truth mask.

With these additions, BG-YOLO11s contains 15.86 million parameters, compared with 10.11 million parameters for the YOLO11s-seg baseline. This increase reflects the added context, boundary, and prototype refinement modules, while keeping the model smaller than several high-capacity segmentation baselines evaluated in this study. The prototype refinement module and the boundary-aware supervision path are shown in Figure 8.

### 3.5. Experimental Setup

All experiments were implemented in Python 3.12 with PyTorch 2.11.0 and the Ultralytics segmentation framework. GPU acceleration used the CUDA 13.0 PyTorch build. Training was performed on a workstation equipped with an NVIDIA GeForce RTX 3090 GPU with 24 GB memory, an Intel Core i9 processor, and 64 GB RAM. All input slices were resized to 512 × 512 pixels before being passed to the network.

Unless otherwise stated, models were trained for 200 epochs with a batch size of 16. Stochastic gradient descent was used with an initial learning rate of 0.01 and cosine learning-rate decay. The default warm-up and weight-decay settings of the Ultralytics framework were retained. YOLO11n-seg, YOLO11s-seg, and YOLO11m-seg were initialised from their corresponding pretrained checkpoints. For the CNN- and Transformer-based baselines, architecture-appropriate settings were used while keeping the input size, data split, training budget, and evaluation protocol consistent across models. The executable configurations, including the installed package build, random-seed setting, framework defaults, custom boundary-loss weight, and Bézier augmentation ranges, are available from the corresponding author, as stated in the Code Availability Statement.

The same 70/15/15 split was used for all experiments, with 2144 training slices, 459 validation slices, and 461 test slices. Bézier Contour Augmentation was applied only to the training set. Two contour-augmented samples were generated for each original training slice, increasing the training pool from 2144 to 6432 images. The validation and test sets were not augmented.

BG-YOLO11s was evaluated against YOLO11 segmentation baselines and representative CNN- and Transformer-based segmentation models. The ablation protocol progressively added dilated context aggregation, boundary-enhanced feature fusion, the prototype refinement module, and Bézier Contour Augmentation to the YOLO11s-seg baseline in a fixed order. This cumulative design measures incremental changes along the selected construction path but does not estimate isolated main effects or all higher-order interactions. The final checkpoint was selected according to validation performance, and all reported metrics were computed once on the held-out test set.

### 3.6. Evaluation Metrics

**Figure 9:**
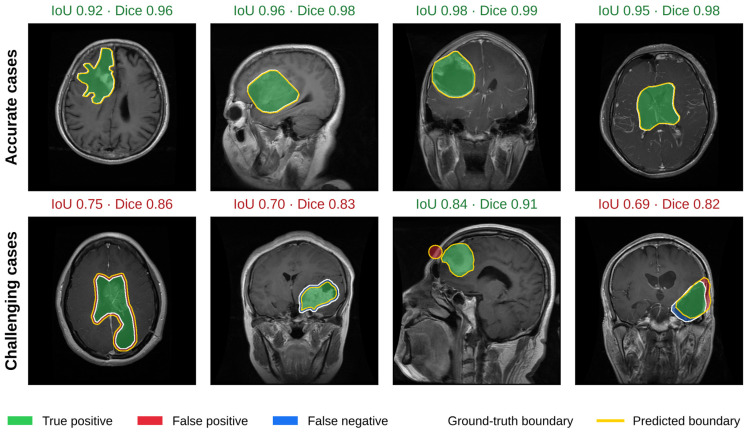
Qualitative segmentation results on the held-out test split. Green regions indicate true-positive overlap, red regions indicate false-positive areas, and blue regions indicate false-negative areas. White and yellow contours show the ground-truth and predicted boundaries, respectively. The examples include accurate segmentations and typical failure patterns, such as mild over-segmentation, under-segmentation, false-positive activation, and small localisation shifts.

Segmentation performance was evaluated on the held-out test set using mask-level precision, recall, mAP@50, mAP@50–95, and IoU. Precision reflects the proportion of predicted tumour instances that correspond to ground truth, while recall reflects the proportion of ground-truth tumour instances successfully detected. IoU quantifies mask overlap, and mAP evaluates mask predictions across confidence levels and IoU thresholds. Dice was additionally calculated for the individual qualitative examples shown in Figure 9, and no aggregate test-set Dice value is reported in Table 3 and Table 4.

Let TP, FP, and FN denote true-positive, false-positive, and false-negative mask predictions at the selected operating threshold. Precision and recall are defined as:

(12) $$Precision=\frac{TP}{TP+FP},$$

(13) $$Recall=\frac{TP}{TP+FN}.$$

For a predicted mask A and a ground-truth mask B, intersection-over-union and the Dice coefficient are computed as:

(14) $$IoU=\frac{\left|A\cap B\right|}{\left|A\cup B\right|},$$

(15) $$Dice=\frac{2\left|A\cap B\right|}{\left|A\right|+\left|B\right|}.$$

Average precision is calculated as the area under the precision–recall curve:

(16) $$AP=\int_{0}^{1}p\left(r\right)\;dr,$$

where p(r) denotes precision as a function of recall. The mAP@50 metric reports mean average precision at an IoU threshold of 0.50, while mAP@50–95 averages AP over IoU thresholds from 0.50 to 0.95 with a step size of 0.05. Since this study uses a single foreground class, the reported mAP corresponds to tumour-mask performance under the same evaluation protocol used for all models.

All metric values are reported as percentages except parameter counts and computational measures. The reported values are single-run point estimates. Confidence intervals, run-to-run standard deviations, and paired significance tests were not computed; therefore, small differences, particularly in the ablation study, should be interpreted cautiously.

## 4. Results

### 4.1. Baseline Comparison

Table 3 presents the test-set performance of the proposed BG-YOLO11s together with representative CNN-, Transformer-, and YOLO-based segmentation models. The comparison includes conventional encoder–decoder models, recent Transformer-oriented segmentation architectures, and three YOLO11 segmentation scales trained under the same dataset split and evaluation protocol. The reported values correspond to mask-level performance on the held-out test set.

Among the non-YOLO baselines, Mask2Former achieved the highest IoU and mAP@50–95, followed by SegFormer and TransU-Net. CNN-based models produced competitive but lower scores, with Attention U-Net performing better than U-Net and DeepLabv3+. The YOLO11 variants showed a clear capacity–performance trend from YOLO11n-seg to YOLO11m-seg. YOLO11s-seg provided a stronger balance than the nanomodel, while YOLO11m-seg improved accuracy at the cost of a larger parameter budget.

BG-YOLO11s produced the highest single-run point estimates across the reported metrics. Compared with the YOLO11s-seg baseline, the proposed model increased precision from 89.1% to 92.4%, recall from 85.0% to 88.7%, mAP@50 from 90.8% to 94.6%, mAP@50–95 from 63.1% to 68.9%, and IoU from 82.4% to 86.5%. These point differences are consistent with improved lesion detection and mask overlap under the fixed image-level benchmark. BG-YOLO11s also produced higher point estimates than YOLO11m-seg while using fewer parameters. Because each configuration was trained once, these results do not establish statistical superiority or isolate the causal contribution of individual modules.

### 4.2. Progressive Ablation Study

Table 4 reports the progressive ablation study. Starting from YOLO11s-seg, the proposed components were added cumulatively in a fixed order. The baseline achieved 90.8% mAP@50, 63.1% mAP@50–95, and 82.4% IoU. Adding dilated context aggregation increased mAP@50–95 to 64.9% and IoU to 83.8%, indicating an incremental improvement along the selected construction path.

Adding boundary-enhanced feature fusion after DCA increased mAP@50–95 to 66.3% and IoU to 84.9%. Adding the prototype refinement module to the DCA+BEFF configuration further increased mAP@50–95 to 67.4% and IoU to 85.6%. Because these configurations are cumulative, the observed changes cannot be interpreted as isolated BEFF-only or PRM-only effects, and possible synergy or redundancy between components is not resolved by the present experiment.

The full BG-YOLO11s configuration, which combined DCA, BEFF, PRM, and Bézier Contour Augmentation, achieved 94.6% mAP@50, 68.9% mAP@50–95, and 86.5% IoU. These stricter overlap measures improved further after BCA was introduced; however, IoU and mAP remain region-overlap metrics and do not directly quantify contour distance. A full factorial, repeated-seed analysis with boundary-specific metrics is required to separate individual module effects from interaction effects.

### 4.3. Qualitative Results

The challenging examples in the lower row of Figure 9 illustrate the principal qualitative failure categories: mild over-segmentation when a thin rim of adjacent enhancing or high-intensity tissue was included, under-segmentation when diffuse, weakly enhanced margins were missed, spurious false-positive activation in visually similar non-tumour regions, and small localisation shifts in low-contrast cases. These categories are descriptive rather than prevalence estimates because a case-level failure taxonomy was not predefined and independently annotated across all 461 test slices.

The qualitative findings are compatible with the overlap-based results, but they do not establish the frequency of each error type. The displayed IoU–Dice pairs are mutually consistent within two-decimal rounding according to Dice = 2IoU/(1 + IoU). The boundary-oriented modules improved mask alignment in the displayed cases, yet they did not eliminate errors caused by low contrast, diffuse margins, and small lesion size. A future case-level audit should assign mutually exclusive failure labels to every test prediction and report their proportions together with boundary F-score, Hausdorff distance, and surface Dice.

## 5. Discussion

This study evaluated whether a YOLO11-based single-stage segmentation model can be made more suitable for brain tumour delineation by adding context-aware, boundary-sensitive, and contour-level components. The motivation came from the structure of the Figshare/Cheng dataset, where most tumour regions occupy a small proportion of the image and where tumour margins often vary in sharpness, shape, and local contrast. These properties make the task difficult for generic instance-segmentation models, because a correct detection is not sufficient when the predicted mask fails to recover the boundary accurately.

The proposed BG-YOLO11s improved the YOLO11s-seg baseline across all reported metrics. The gain was particularly clear in mAP@50–95 and IoU, which impose stricter overlap requirements than mAP@50 but are not dedicated boundary-distance measures. Compared with YOLO11s-seg, BG-YOLO11s increased mAP@50–95 from 63.1% to 68.9% and IoU from 82.4% to 86.5%. The improvement over YOLO11m-seg is also noteworthy because the proposed model achieved higher point estimates with fewer parameters than the larger YOLO11 variant. Nevertheless, these comparisons are based on one image-level split and one training run, so statistical superiority is not claimed.

The progressive ablation indicates that performance increased as DCA, BEFF, PRM, and BCA were added along the selected construction path. This pattern is compatible with benefits from wider context, boundary-sensitive fusion, prototype refinement, and contour variation. However, the cumulative design does not provide BEFF-only, PRM-only, BCA-only, or every pairwise and higher-order combination. It, therefore, cannot distinguish independent main effects from synergy or redundancy, and the single-run differences may also contain optimisation variance.

The comparison with CNN- and Transformer-based baselines supports the potential value of problem-specific model design under the present benchmark. Classical encoder–decoder models provide strong spatial reconstruction, Transformer-oriented models improve contextual interaction, and volumetric approaches exploit inter-slice information [17]. In this study, BG-YOLO11s produced the highest single-run mask-level point estimates with a moderate parameter count. This does not establish that YOLO-based models are generally superior, because the evaluation is limited to one image-level split of one T1-CE dataset. Contemporary BraTS glioma protocols instead use multi-institutional and multi-sequence evaluation conditions [18]. Related BraTS-METS evaluation extends this stronger validation standard to brain metastases [19].

Table 5 places the proposed model in the context of representative brain tumour segmentation studies. The comparison should be interpreted cautiously because previous studies differ in dataset version, patient split, preprocessing, dimensionality, MRI sequences, tumour-class formulation, supervision type, and reported metrics. Table 5 is, therefore, not a direct ranking. Its purpose is to clarify methodological differences and to show that the strongest quantitative evidence in this study remains the internal, single-run comparison reported in Table 3 and Table 4.

The qualitative results identify plausible residual error modes, including over-segmentation along enhancing rims, under-segmentation at diffuse boundaries, false-positive activation in visually similar regions, and small localisation shifts. Because the test cases were not prospectively assigned failure labels, the manuscript does not report percentages for these categories. This prevents a prevalence claim and reinforces the need for a complete case-level audit and boundary-specific metrics, since IoU and mAP do not fully describe clinically relevant contour errors.

The findings should, therefore, be read as preliminary evidence for boundary-aware YOLO11 segmentation under a controlled image-level benchmark protocol, rather than as evidence of patient-level generalisation or clinical readiness. Broader claims depend on patient-disjoint retraining, repeated-seed statistics, explicit boundary evaluation, external multi-centre testing, and multi-sequence analysis.

## 6. Limitations and Future Work

The main limitation is the image-level split. The exported image-and-polygon dataset used for training did not preserve patient identifiers, so slices from the same patient may occur in more than one subset. Patient-level leakage, therefore, cannot be excluded. Recovering patient identifiers from the original MAT-file repository and repeating every baseline and ablation run is necessary to establish performance on unseen patients.

A second limitation is evaluation on a single public dataset and a single MRI sequence. The Figshare/Cheng data do not represent the full heterogeneity of scanners, field strengths, acquisition protocols, reconstruction settings, institutions, and patient populations. No independent external cohort or T1/T2/FLAIR multi-sequence experiment was performed; therefore, cross-domain and multi-sequence generalisation are unknown.

The task was formulated as single-class tumour segmentation. It does not distinguish meningioma, glioma, and pituitary tumour during prediction and, therefore, does not provide histology-aware segmentation or joint segmentation–classification. In addition, Bézier Contour Augmentation changes contour morphology but does not create new tumour texture, enhancement patterns, or scanner-dependent intensity variation.

The highest-priority future experiment is a strict patient-disjoint split reconstructed from the original patient records, followed by retraining of all baselines and ablations. Independent cohorts from different institutions and scanners should then be used for external validation. Multi-sequence inputs incorporating T1, T1-CE, T2, and FLAIR would determine whether the boundary-guided design transfers beyond contrast-enhanced T1-weighted slices.

Further evaluation should include three to five independent seeds, mean ± standard deviation, confidence intervals, and paired tests performed on matched test cases. A full factorial ablation should include isolated and combined DCA, BEFF, PRM, and BCA configurations. Boundary F-score, HD95, and surface Dice should be reported together with a complete 461-case failure audit. The framework should also be extended to multi-class segmentation or joint segmentation–classification, and efficiency should be profiled using FLOPs, latency, throughput, memory use, and device-level measurements.

## 7. Conclusions

This study presented BG-YOLO11s, a boundary-guided YOLO11-based instance-segmentation model for brain tumour delineation in contrast-enhanced T1-weighted MRI. The model extends YOLO11s-seg with dilated context aggregation, boundary-enhanced feature fusion, and a prototype refinement module trained with explicitly defined differentiable morphological gradient supervision. Bézier Contour Augmentation was introduced to increase contour-level variability while preserving surrounding anatomy. On the fixed image-level Figshare/Cheng test split, BG-YOLO11s achieved 92.4% precision, 88.7% recall, 94.6% mAP@50, 68.9% mAP@50–95, and 86.5% IoU in a single run. The progressive ablation showed increasing point estimates along the selected module-addition path, but it did not isolate all module interactions or quantify run-to-run uncertainty. The current evidence is, therefore, limited to an image-level, single-dataset, single-sequence benchmark. Patient-disjoint retraining, repeated-seed statistical analysis, boundary-specific metrics, complete failure pattern quantification, external multi-centre validation, and multi-class or multi-sequence experiments are required before broader clinical or deployment-oriented conclusions can be drawn.

## Figures and Tables

**Table 3:** Test-set segmentation performance of representative CNN-, Transformer-, and YOLO-based models together with the proposed BG-YOLO11s. All metric values are percentages and are reported as single-run point estimates. Bold values indicate the best result in each metric column.

Model	Type	Params (M)	Mask P	Mask R	mAP@50	mAP@50–95	IoU
U-Net	CNN	31.0	83.9	80.2	84.8	55.4	76.5
DeepLabv3+	CNN	39.8	85.6	82.1	86.7	57.9	78.3
Attention U-Net	CNN	34.9	86.0	82.7	87.4	58.7	79.0
TransU-Net	Transformer	105.3	86.8	83.5	88.3	60.1	80.3
Swin-U-Net	Transformer	27.2	86.5	83.2	88.0	59.7	79.8
SegFormer (MiT-B2)	Transformer	27.5	87.1	84.0	88.7	60.9	80.7
Mask2Former (Swin-T)	Transformer	47.0	87.8	84.6	89.4	62.0	81.6
YOLO11n-seg	YOLO	2.88	86.4	82.1	87.6	58.3	78.6
YOLO11s-seg	YOLO	10.11	89.1	85.0	90.8	63.1	82.4
YOLO11m-seg	YOLO	22.42	90.0	86.2	91.7	64.8	83.5
**BG-YOLO11s (ours)**	**YOLO**	**15.86**	**92.4**	**88.7**	**94.6**	**68.9**	**86.5**

**Table 4:** Progressive, cumulative module-addition study on the held-out image-level test split. All values are single-run percentages—the experiment is not a full factorial ablation. Metrics are mask-level single-run percentages. Configurations are cumulative and do not represent isolated or full factorial module effects. ✓ indicates that the module was included in the configuration, and – indicates that it was not. Bold values indicate the best result in each metric column.

Configuration	DCA	BEFF	PRM	BCA	mAP@50	mAP@50–95	IoU
Baseline (YOLO11s-seg)	–	–	–	–	90.8	63.1	82.4
+ DCA	✓	–	–	–	92.0	64.9	83.8
+ DCA + BEFF	✓	✓	–	–	93.1	66.3	84.9
+ DCA + BEFF + PRM	✓	✓	✓	–	93.8	67.4	85.6
**BG-YOLO11s (full + BCA)**	**✓**	**✓**	**✓**	**✓**	**94.6**	**68.9**	**86.5**

Metrics are mask-level single-run percentages. Configurations are cumulative and do not represent isolated or full factorial module effects.

**Table 5:** Indicative comparison of BG-YOLO11s with representative brain tumour segmentation studies using the Figshare/Cheng dataset or closely related T1-CE MRI data. NR indicates that the exact value was not reported or was not directly comparable in the cited study.

Method	Dataset	Model Type	Main Methodological Focus	Reported Result	Relation to the Present Study
Zheng et al. [7]	Figshare/Cheng	Improved U-Net	Hybrid dilated convolutions and Dice-based optimisation	Improved Dice over vanilla U-Net	Addresses multi-scale context, but does not explicitly evaluate contour-level augmentation or YOLO-based prototype refinement
YOLO-BT [12]	T1-CE MRI	YOLO-based segmentation	YOLOv11-based tumour segmentation for irregular shapes and size variation	mIoU +6.1%, Dice +3.6% over baseline	Closely related YOLO-based work, but does not combine DCA, BEFF, PRM, and Bézier Contour Augmentation
U-Net/FCN/YOLO hybrid study [13]	BTS-DS 2024	CNN and YOLO benchmarking	Comparative evaluation of U-Net, FCN, and YOLO variants	YOLOv9e: F1 92.25%, IoU 85.62%	Supports the relevance of YOLO-style models, but uses a different dataset
Two-headed U-Net-EfficientNet [14]	Figshare/Cheng	Multi-task encoder–decoder	Parallel segmentation and classification	NR	Combines segmentation and classification, but follows a multi-task architecture rather than a compact YOLO-style segmentation pipeline
QuickTumorNet [15]	Cheng 2D MRI	Fast CNN segmentation	Fast multi-class tumour segmentation	NR	Emphasises speed and multi-class segmentation, but explicit boundary-guided mask refinement is not the main focus
U-Net with ResNet-18 and DenseNet [16]	Figshare/Cheng	Two-stage CNN pipeline	U-Net-based localisation followed by DenseNet classification	91–97% classification accuracy after segmentation	Mainly classification-oriented after segmentation, so mask-level comparability is limited
Ramzan et al. [17]	Volumetric MRI	3D CNN	Volumetric brain region segmentation using inter-slice context	Reported in the original study	Shows the value of 3D context, but is not directly comparable with 2D single-stage tumour instance segmentation
BraTS glioma challenge [18]	Multi-institutional, multi-sequence MRI	Challenge benchmark	Patient-disjoint glioma segmentation with lesion-wise Dice and HD95	Challenge-specific results	Provides a stronger generalisation and boundary evaluation standard than the present image-level T1-CE benchmark
Weakly supervised XAI study [20]	Cheng/BraTS	Explainability-based segmentation	Segmentation evidence derived from classification models	NR	Reduces dense annotation dependence, but provides less direct control over boundary precision
BG-YOLO11s, this study	Figshare/Cheng	Boundary-guided YOLO11	DCA, BEFF, PRM, boundary-aware supervision, and Bézier Contour Augmentation	mAP@50 94.6%, mAP@50–95 68.9%, IoU 86.5%	Evaluated internally against baselines trained under the same split and protocol

## Data Availability

The Figshare brain tumour dataset analysed in this study is publicly available at: https://doi.org/10.6084/m9.figshare.1512427.v5. The source code, model definitions, executable training and evaluation configurations, and trained weights are available from the corresponding author upon reasonable request. The MRI data are not redistributed—users should obtain them from the original Figshare repository.
